# Search for HRV-parameters that detect a sympathetic shift in heart failure patients on β-blocker treatment

**DOI:** 10.3389/fphys.2013.00081

**Published:** 2013-04-16

**Authors:** Yanru Zhang, Olav R. de Peuter, Pieter W. Kamphuisen, John M. Karemaker

**Affiliations:** ^1^AEF/Systems Physiology, Heart Failure Research Center, Academic Medical Center at the University of AmsterdamAmsterdam, Netherlands; ^2^Vascular Medicine, Academic Medical Center at the University of AmsterdamAmsterdam, Netherlands

**Keywords:** intensive care, home monitoring, heart rate variability, time domain analysis, frequency domain analysis, entropy

## Abstract

**Background**: A sympathetic shift in heart rate variability (HRV) from high to lower frequencies may be an early signal of deterioration in a monitored patient. Most chronic heart failure (CHF) patients receive β-blockers. This tends to obscure HRV observation by increasing the fast variations. We tested which HRV parameters would still detect the change into a sympathetic state.

**Methods and results**: β-blocker (Carvedilol®) treated CHF patients underwent a protocol of 10 min supine rest, followed by 10 min active standing. CHF patients (NYHA Class II–IV) *n* = 15, 10m/5f, mean age 58.4 years (47–72); healthy controls *n* = 29, 18m/11f, mean age 62.9 years (49–78). Interbeat intervals (IBI) were extracted from the finger blood pressure wave (Nexfin®). Both linear and non-linear HRV analyses were applied that (1) might be able to differentiate patients from healthy controls under resting conditions and (2) detect the change into a sympathetic state in the present short recordings.

**Linear**: mean-IBI, SD-IBI, root mean square of successive differences (rMSSD), pIBI-50 (the proportion of intervals that differs by more than 50 ms from the previous), LF, HF, and LF/HF ratio.

**Non-linear**: Sample entropy (SampEn), Multiscale entropy (MSE), and derived: Multiscale variance (MSV) and Multiscale rMSSD (MSD). In the supine resting situation patients differed from controls by having higher HF and, consequently, lower LF/HF. In addition their longer range (τ = 6–10) MSE was lower as well. The sympathetic shift was, in controls, detected by mean-IBI, rMSSD, pIBI-50, and LF/HF, all going down; in CHF by mean-IBI, rMSSD, pIBI-50, and MSD (τ = 6–10) going down. MSD6–10 introduced here works as a band-pass filter favoring frequencies from 0.02 to 0.1 Hz.

**Conclusions**: In β-blocker treated CHF patients, traditional time domain analysis (mean-IBI, rMSSD, pIBI-50) and MSD6–10 provide the most useful information to detect a condition change.

## Introduction

Heart rate variability (HRV) analysis has seen an increasing interest since the early work of B.McA. Sayers in the 1970's (Hyndman et al., 1971), picking up speed since the 1980's (Akselrod et al., 1981; Baselli et al., 1987; deBoer et al., 1987). However, these analysis techniques still have not made it to the bedside, probably due to the fact that equipment manufacturers do not offer standard solutions in ECG-monitors to provide intricate HRV-data. Only recently, some HRV-analysis methods have sneaked into the clinic by a “stealth” method, hidden in algorithms that give a generalized “alert value” to a patient condition, based on observation of a series of vital parameters (Clark et al., 2012; Lee et al., 2012; Morris et al., 2007).

In the present study we tried a clinical approach to a problem that has received little attention in biomedical literature. It has been fairly well-established that chronic heart failure patients (CHF) have different HRV patterns compared to matched healthy controls (Saul et al., 1988; Casolo et al., 1989). However, many of those patients will be on β-blocker therapy, which has a strong influence on both HR and HRV (Coumel et al., 1991; Pousset et al., 1996; Mortara et al., 2000; Kubo et al., 2005). Under these circumstances HRV has been shown to improve toward normal (Mortara et al., 2000; Lin et al., 2001), a fact that might mask an underlying developing disease status. We therefore tested which HRV-analysis technique might be able to give early warning when there is a “slipping of” in the sympathetic direction of the autonomic balance. To test this we used recordings in CHF patients who went from supine to upright, as a simple model to reduce vagal outflow to the heart and induce generalized sympathetic activation. For this study we had a set of 21 recordings in CHF-patients on Carvedilol® treatment that has been fully described earlier (Truijen et al., 2011). In view of the practical applicability in situations where a diagnosis should be available after a short period of recording only, we were wondering if the 10 min in supine posture followed by 10 min upright that we had were sufficient to answer two questions:

1. Is there still a distinction health/disease when comparing the supine recordings in β-blocker treated CHF patients to those from matched, healthy controls?
2. Can HRV-analysis demonstrate an intra-individual shift toward a more sympathetic state when comparing the upright to the supine recording, even in this group of β-blocker treated patients?

The first question is of importance for a quick triage of patients, the second to detect a deterioration of a patient's health before a “normal” alarm would sound.

We decided to test a number of obvious linear measures, from the time domain: mean-IBI (interbeat interval), SD-IBI (standard deviation), rMSSD (root mean square of successive differences of IBI's), pIBI-50 (proportion of pairs of successive IBI's that differ by more than 50 ms) and from the frequency domain: LF, HF and LF/HF (Low Frequency around 0.1 Hz, High Frequency, i.e., respiratory frequency, mostly around 0.25 Hz, and their quotient). In view of earlier studies where the use of short recordings for non-linear analysis has been analyzed (Batchinsky et al., 2009; Lake and Moorman, 2011; Leistedt et al., 2011; Turianikova et al., 2011), we decided to use SampEn [sample entropy (Richman and Moorman, 2000)] and Multiscale Entropy (MSE; Costa et al., 2002). The latter method computes entropy over progressively coarser grained versions of the original series. As a by-product we considered the variances (Multiscale variance or MSV) and multiscale root mean square of successive differences (MSD) of those newly constructed series as well.

A successful analysis method or combination of methods should be able to do the triage (Question 1) as well as detect the sympathetic shift (Question 2) within the limits of the 20 min recordings that were available.

## Methods

### Study population

#### Patients

The patient recordings had been made in the study that has extensively been described in (Truijen et al., 2011). In short: 21 CHF patients (NY Heart Assoc. classification II–IV) participated in a study that was directed to discrimination of β-blocker sensitivity depending on the specific β_2_-receptor subtype that was present in the patient. In a double-blind cross-over design they received the non-selective β-blocker Carvedilol® (Eucardic, Roche, Mijdrecht, Netherlands) or the selective metoprolol succinate (Selokeen ZOC, AstraZeneca, Zoetermeer, Netherlands) as β-blocker for 6 weeks. Both drugs were titrated to equipotent dosages, additionally checked by resting heart rate. Since recordings made under Carvedilol showed fewer extrasystoles and other rhythm disturbances, we have restricted our study to the recordings made after 6 weeks on this drug. It should be mentioned here that Carvedilol is known to also have α_1_-blocking properties, without intrinsic sympathetic activity (Eggertsen et al., 1984).

Patients had given their written informed consent after study approval by the local Ethics committee. Due to problems with too frequent premature ventricular contractions and erroneous blood pressure tracings, 6 out of the original 21 patient recordings (Truijen et al., 2011) under Carvedilol had to be rejected. This left 15 CHF patients for the present study, 10/5 (male/female), age 58.4 ± 6.5 (mean ± SD), BMI 27.4 ± 6.0.

#### Healthy control subjects

Subjects had been recruited by advertisement and selected to match the patient group by gender, age and β_2_-receptor subtype. They were in good health, free of cardiovascular disease, non-smokers. After written, informed consent 34 subjects participated. Due to technical problems in the recordings and 2 cases of near-syncope in the stand-test, 5 out of the original 34 control recordings had to be rejected. This left 29 (18/11, m/f), age 62.9 ± 7.3 BMI 26.1 ± 4.2 for analysis. There are no significant differences in age and BMI distribution between healthy controls and CHF patients.

### Measurements and data preprocessing

Continuous non-invasive blood pressure was measured from a finger by the volume-clamp technique. A Nexfin® (BMEYE, Amsterdam, Netherlands) hemodynamic monitor was used with instantaneous display of reconstructed upper arm blood pressure, heart rate, pulse contour derived cardiac output and systemic vascular resistance. This enabled proper monitoring during the stand test. To prevent hydrostatic errors the hand was held at heart level in both positions by a sling around the neck.

Patients and controls underwent a test protocol which included blood draws for clotting factors in the supine position as described earlier (Truijen et al., 2011). Then they rested for at least 20 min before actively standing up. They remained standing for another 10 min. From the Nexfin computed data we only analysed IBI values for the present study, measured to an accuracy of 5 ms (200 Hz sample rate of A/D conversion).

In view of dysrhythmias like PVC's, other rhythm disturbances and, occasionally, movement artifacts that were present in the IBI-recordings, they had to be pre-processed before analyses could be performed. We used a two-step spike-removal procedure. First we established the global mean value IBI_mean−glb_ of the whole set (supine or upright), and substituted any IBI_i_ outside the range 80–120% of IBI_mean−glb_ by that value. Next, a 10-beat window would slide over the recording, replacing any newly added IBI_j_ outside the 80–120% range around the IBI_mean−local_ by the value of the local mean. The first step deletes sharp spikes globally, making it easier for the second step which is required to preserve continuity of the time series.

### Calculation of HRV parameters

#### Linear methods

Since we derived heart periods from blood pressure recordings rather than from an ECG, we cannot call them NN-intervals (normal to normal) since, strictly spoken, we have no information on the origin of the heartbeat, whether it originates from the sinus node or from some other pacemaking site in the heart. Although all patients underwent a test-ECG just prior to the present recording, where normal sinus rhythm had been established, we will use the more general term “IBI” (interbeat interval) instead.

After data pre-processing as described above we calculated mean-IBI, SD-IBI, rMSSD, pIBI-50 (the proportion of intervals that differs by more than 50 ms from the previous) following the usual methods (Task Force of the European Society of Cardiology, and the North American Society of Pacing and Electrophysiology, 1996). We chose a period of at least 5 min stable recording for both the supine and upright periods. Of the upright recording a period of 2 min after the standing up maneuver was skipped, to allow for the first transient in blood pressure and heart rate to disappear. This left a period of maximally 8 min upright to be included in the computations.

For the frequency analysis we used the IBI data set without interpolation, putting the average interbeat interval as spacing between heart beats (deBoer et al., 1984). After removal of a linear trend and Hanning-windowing we applied a digital Fourier transform (Matlab®) rather than FFT. This method can be applied to an arbitrary number of data points without the need of zero-padding until a power of two has been reached. LF, HF, and LF/HF ratio were computed after integration of the spectral curve from 0.04 to 0.15 Hz for LF and from 0.15 to 0.4 Hz for HF. The values were reduced to normalized units by division by the total variance (Task Force, 1996).

#### Non-linear methods: SampEn and MSE

The calculation of MSE has been fully described in (Costa et al., 2005). It is the sample entropy (SampEn) (Richman and Moorman, 2000) of consecutively coarser grained time series Y constructed from the original time series X:{x_1_,…, x_i_,…, x_N_} by a scale factor of τ.

**Figure 1:**
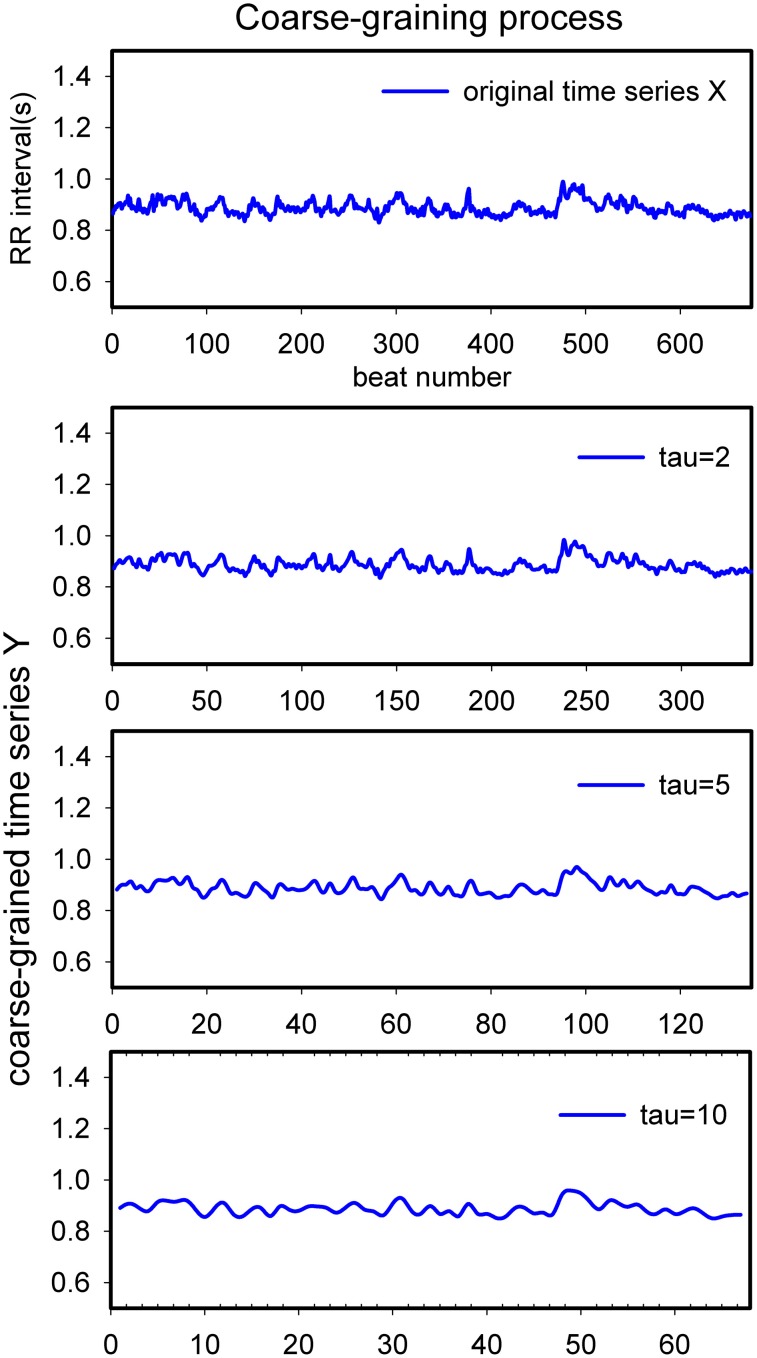
**Schematic diagram of the process of coarse-graining.** This is a representative ~10 min heart rate recording from a healthy volunteer in supine position. From top to bottom tau = 1, 2, 5, 10. X-scale: item number in the series; Y-scale: (averaged) duration of heart periods in seconds.

The coarse-graining procedure is the first step to compute MSE, as well as MSV and multiscale successive differences (MSD). By taking τ consecutive values together, the original signal is progressively “smoothed” and more and more beat-to-beat “noise” is averaged out. This process is visualized in Figure 1 and formalized in formula 1:

(1) $$y_{j}^{\left(\tau \right)}=\frac{1}{\tau }\sum_{i\;=\;(j\;-\;1)\tau \;+\;1}^{j\tau }x_{i},\;1\leq j\leq N/\tau .$$

This describes a set of consecutively more coarse-grained time series, [y^(τ)^] from the series X, where τ is the scale factor. Next, the SampEn (Richman and Moorman, 2000) of each time series[y(τ)] is computed, resulting in MSE. SampEn is a measure of the probability that a sequence of m consecutive data points will not remain similar (within a given tolerance *r*) at the next point in the data set. A high SampEn value implies low regularity, i.e., few repetitions of the same pattern. Details on how to calculate SampEn can be found in references (Richman and Moorman, 2000; Costa et al., 2002; Xinnian et al., 2006).

In short, MSE aims to measure the complexity of the system. In a totally random sequence, SampEn will decrease to zero with increasing τ; in a sequence that has been generated by a system with some degree of complexity, like heart rate over time, it tends to find a stable non-zero value.

#### Linear extension: MSV and MSD

We also computed a side-product of the coarse-graining process, i.e., the (multiscale) variance (MSV) and multiscale rMSSD (MSD) of the newly constructed series Y(τ). We reasoned that these might show in a simple way the variability of heart rate at medium-scale time-intervals.

### Statistics

All computations were done by use of SPSS®. After testing for normality, comparisons between groups were done by pairwise testing using Student's *t*-test or Mann–Whitney *u*-test where appropriate. For the change to a sympathetic state within one subject, we used the computed supine value to normalize the upright value. The resulting quotient upright/supine was then linearized by a log-transformation before statistical testing.

To compensate for the multiple comparisons we adapted the test magnitude alpha by applying the Bonferroni–Holm correction. This will lead to a value of alpha smaller than the 0.05 that we considered significant. The corrected alpha is mentioned in the tables along with the computed exact *p*-value. This allows the reader to judge the significance of observed changes, taking into account the type I/type II error as well as the biological significance of the change (Cabin and Mitchell, 2000; Nakagawa, 2004).

## Results

### Comparison CHF patients vs. healthy controls

Table 1 gives an overview of the chosen 11 HRV parameters in patients and control subjects in the supine posture. It is remarkable that the mean supine heart rates, pIBI-50 and rMSSD in the two groups are equal despite the β-blockade in the patients. Total variability as expressed by SD-IBI is lower in CHF, although not statistically significant. HF as short term variability index is higher in CHF, but this may well be due to the β-blockade (Goldsmith et al., 1997; Witte and Clark, 2008). As a consequence LF/HF is significantly lower in CHF as well.

**Table 1:** **Parameter comparison between control subjects and CHF patients in 10-min supine posture**.

**Parameter (units)**	**Control**	**CHF**	***p*-value**	**Bonferroni–Holm corrected alpha**	**Deemed significant**
meanIBI (ms)	1002 ± 158	951 ± 120	0.281	0.02	no
SD-IBI (ms)	38.0 (21.8–87.2)	32.4 ± 15.7	0.090	0.01	no
rMSSD (ms)	25.7 (12.1–125.4)	27.8 ± 14.5	0.795	0.05	no
pIBI-50 (proportion)	0.04 (0.00–0.84)	0.03 (0.00–0.26)	0.586	0.025	no
LF (n.u.)	0.19 ± 0.07	0.13 ± 0.08	0.025^*^	0.007	no
HF (n.u.)	0.25 (0.09–0.72)	0.52 ± 0.23	0.000^#^	0.005	yes
LF/HF	1.06 ± 0.73	0.36 ± 0.36	0.000^#^	0.005	yes
SampEn	1.51 ± 0.37	1.69 ± 0.32	0.152	0.0125	no
MSE_6–10_	1.57 ± 0.21	1.35 ± 0.39	0.015^*^	0.006	yes
MSV_6–10_ (ms^2^)	870 (303.4–4210.4)	554 (3.0–1880.8)	0.046^*^	0.008	no
MSD_6–10_ (ms)	24.5 (13.6–75.2)	19.6 ± 11.0	0.016^*^	0.006	no

*If data are normal distributed, the value is as mean ± std and the Student t-test is applied; if non-normal distributed, the value is as median (minimum–maximum) and the Mann–Whitney U test is applied. Before Bonferroni–Holm correction*,

* p < 0.05;

# *p < 0.01; n.u. = normalized units (power in the respective bands is normalized by division by total variance). The values of MSE, MSV, and MSD are computed by averaging over the 5 highest tau values: sum[MSE(tau = 6:10)]/5, sum[MSV(tau = 6:10)]/5, sum[MSD(tau = 6:10)]/5. The column “deemed significant” gives the interpretation of the authors, taking the p-value, corrected alpha and the biological significance into account; cf. text*.

**Figure 2:**
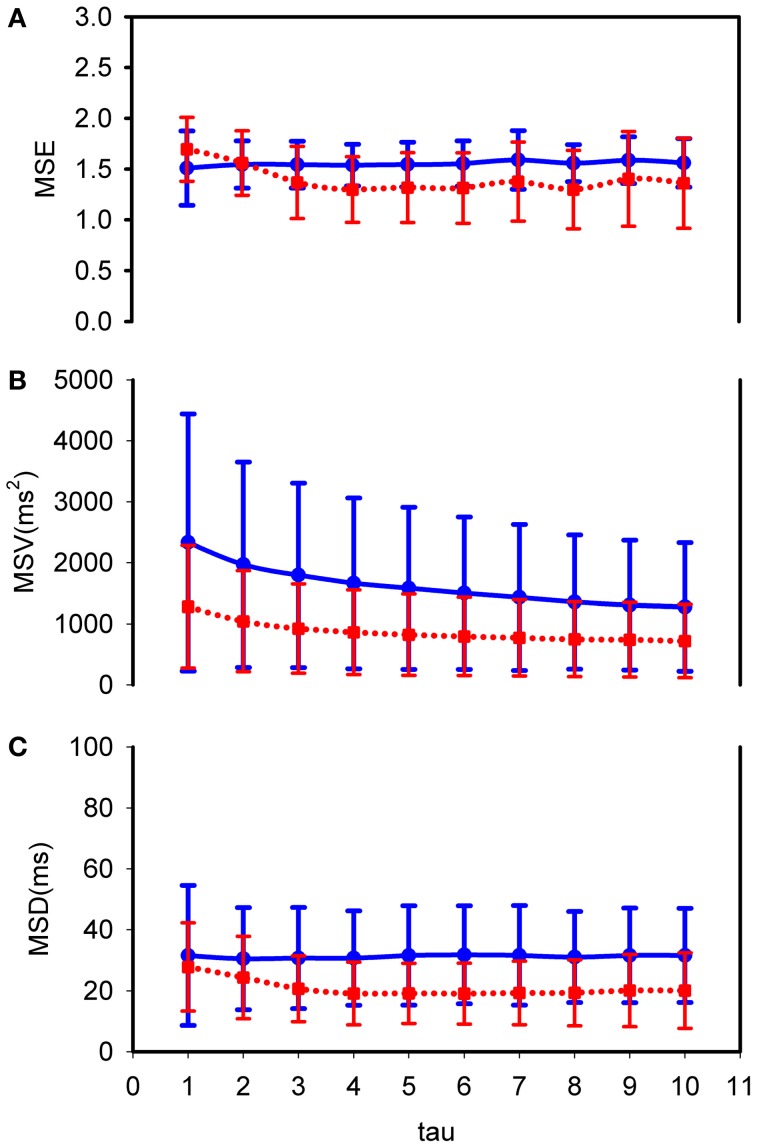
**MSE, MSV, and MSD curves: comparison between control subjects and CHF patients in supine posture. (A)** MSE; **(B)** MSV; **(C)** MSD. Fat (blue) line: control healthy subjects; dotted (red) line: CHF patients. Tau from 1 to 10. The curves represent mean values with ±1 standard deviation. CHF patients have lower MSE, MSV, and MSD than healthy subjects for tau above 2.

To further analyze the internal structure of the variability we computed, first, the MSE-curves for τ = 1–10, results shown in Figure 2A. At τ = 1 SampEn in CHF and controls are equal (Table 1). For values of τ above 3 the curve of the CHF-patients falls below that of the healthy controls. However, a large overlap exists. To emphasize the longer range interactions rather than the short-term variability (Ho et al., 2011) we integrated the values for τ from 6 to 10, resulting in the MSE_6−10_ number in Table 1. The variances of the coarse grained distributions for increasing τ are depicted by the MSV as shown in Figure 2B. Average MSV in CHF is lower than that in controls for all values of τ; we averaged the value for τ= 6–10 as MSV_6−10_ in Table 1. Except for τ = 1 the same holds true for the rMSSD of the coarse grained distributions, expressed as MSD in Figure 2C and averaged to one value from τ = 6 to 10 in Table 1. Both MSV_6−10_ and MSD_6−10_ are lower in CHF than in controls, however, in view of the wide distribution of the numbers, taking the Bonferroni–Holm corrected alpha and the magnitude of the difference into account, we do not consider these differences biologically significant. The same cannot be said for MSE_6−10_: although the number fails to meet the Bonferroni–Holm corrected alpha (*p* = 0.05/9 = 0.006), yet in view of the narrow distribution and the exact *p*-value of 0.015 we do consider this difference significant.

### Sympathetic shift in control subjects: upright vs. supine posture

We computed the 11 parameters again for the upright condition, and expressed the upright value as fraction of the individual supine one. For statistical testing the values have been log-transformed, to obtain values which follow a normal distribution with mean = 0 if the supine and upright values are equal. The averages and *p*-values are shown in Table 2.

**Table 2:** **Control subjects and CHF patients: normalized parameters (=upright/supine)**.

**Normalized Parameter = (upright/supine)**	**Control subjects**	***p*-value**	**CHF patients**	***p*-value**
Mean IBI	0.84 ± 0.06	0.000^#^	0.90 ± 0.06	0.00003^#^
SD-IBI	0.98 ± 0.36	0.253	0.92 ± 0.44	0.130
rMSSD	0.72 ± 0.28	0.00001^#^	0.69 ± 0.24	0.0003^#^
pIBI-50	0.36 (0.00–1.75)	0.000^#^	0.15 (0.00–2.55)	0.009^#^
LF	1.60 ± 1.65	0.123	1.19 ± 0.92	0.635
HF	0.89 ± 0.55	0.019^*^	1.00 ± 0.71	0.279
LF/HF	2.87 ± 3.36	0.008^#^	2.01 ± 2.91	0.723
SampEn	0.96 ± 0.30	0.066	0.94 ± 0.29	0.199
MSE_6–10_	1.02 ± 0.18	0.256	0.98 (0.31–1.63)	0.609
MSV_6–10_	1.39 ± 1.17	0.989	1.55 ± 2.42	0.540
MSD_6–10_	1.01 ± 0.48	0.314	0.76 ± 0.22	0.001^#^

*If data are normal distributed, the value is as mean ± sd and a one-sample t-test is applied; if non-normal distributed, the value is as median (minimum–maximum) and a Wilcoxon signed-rank test is applied. Normalized parameters are calculated as: (value of upright)/(value of supine) for every individual. After log-transformation a one-sample t-test has been used to test the deviation from zero (i.e., upright value = supine value)*;

* p < 0.05;

# *p < 0.01 (within-group differences for upright to supine); the values of MSE, MSV, and MSD are as in Table 1: sum[MSE(tau = 6:10)]/5, sum[MSV(tau = 6:10)]/5, sum[MSD(tau = 6:10)]/5*.

As to be expected, mean-IBI has significantly decreased, i.e., heart rate goes up on standing. All short-term variability measures go down as well: rMSSD, pIBI-50 and HF. The increase in LF did not reach statistical significance; LF/HF increased in line with the decreased HF. SampEn and the coarse grained measures, MSE_6−10_, MSV_6−10_, and MSD_6−10_ did not change.

### Sympathetic shift in CHF patients: upright vs. supine posture

Generally speaking, the sympathetic change in CHF-patients followed the same pattern as in healthy control subjects, without statistically significant differences between the two groups. However, there were a few notable within-group exceptions, as shown in Table 2.

In the patients mean-IBI decreased significantly, so did rMSSD, pIBI-50, but none of the frequency analysis measures. Of the non-linear and coarse-grained parameters only MSD_6−10_ decreased significantly, the other ones did not change appreciably.

### Absolute power results from fourier analysis

Fourier analysis of HRV may be analyzed in many ways; we chose to look at the individual normalized powers of LF and HF and the LF/HF quotients. However, if the underlying (absolute) data are very much changed by the intervention (standing up in this case) these numbers may be misleading. Therefore, we checked the absolute powers and in addition those in the VLF band (very low frequency band: from the lowest observed frequency in the 5–10 min recording to 0.04 Hz). The results are given in Table 3. No significant changes in total power or power in the various bands with standing were detectable. These might have invalidated the above analyses.

**Table 3:** **Total power and power in the various bands: VLF, LF, HF; supine values compared to upright**.

**Parameter (ms^2^)**	**Control subjects [median (min–max)]**		**CHF patients**	
	**Supine**	**Upright**	**Supine**	**Upright**
Total power	1445 (474–7602)	1437 (226–9627)	1162 (44–2974)	677 (59–2823)
VLF	770 (271–4477)	934 (133–5147)	265 (1–2106)	322 (0–2086)
LF	243 (34–1804)	235 (43–3185)	116 (6–646)	75 (2–537)
HF	344 (44–4902)	189 (49–1295)	464 (27–1561)	238 (57–1036)

In view of the non-normal distributions of absolute powers the medians and ranges are given.

No within-group significant changes from supine to upright can be demonstrated due to the large variability.

## Discussion

The present computer-based *post-hoc* study tried to establish the numbers that could aid in fast diagnosis of a β-blocker treated patient's “slipping off” into a more sympathetic state. We chose a stand-test as model for this condition; no one can stand very well for 10 min without sympathetic system involvement in view of the induced drop in blood pressure at the level of the carotid sinuses and the relative hypovolemia that is observed by pressure sensitive receptors in the low-pressure area (atria, lungs) (Borst et al., 1982; Ten Harkel et al., 1993).

We reasoned that patients on a β-blocker, when remotely monitored or admitted to an intensive care unit for acute exacerbation of symptoms, might pose additional challenges to a monitoring system that would incorporate HRV-measures in an intelligent alarm. Beta-blockers have a tendency to increase short term HRV as well as total background variability as it may be observed in the low to very low frequency ranges (Goldsmith et al., 1997; Aronson and Burger, 2001; Bullinga et al., 2005). Moreover, the additional α_1_-blocking properties of Carvedilol may lead to less apparent blood pressure waves when sympathetic arousal takes place. This property has been pointed out as instrumental in not lowering HR as much as do other β-blockers (Stoschitzky et al., 2001), as compensation for the decreased systemic resistance that it provokes (Ferrua et al., 2005).

In line with our initial suppositions we found that in the supine resting state the CHF-patients differed from the healthy control subjects by showing equal HR with almost equal SD-IBI, but significantly higher HF variability, therefore lower LF/HF ratio. Furthermore, the patients had slightly lower values for MSE_6−10_, MSV_6−10_, and MSD_6−10_. In our view this is mirroring the increased beat-to-beat variability due to the β-blocker together with a slightly increased sympathetic activation. When going to the upright posture the control subjects displayed most of the expected changes: increased HR, decreased rMSSD, pIBI-50, HF, increased LF/HF, but not an increased LF, probably due to the large variance in this measure. MSE_6−10_, MSD_6−10_, or MSV_6−10_ did not record a change. When it came to the CHF-patients in upright posture the parameters that did show up as useful were HR (increased), rMSSD, pIBI-50 and MSD_6−10_ (decreased). None of the other parameters would indicate a shift into a sympathetic state. Our results in the healthy control group tally well with those of Turianikova et al. (2011) who recently published a comparable orthostasis study. Exception is our lack of results for MSE_6−10_; in comparison we would have expected a definite increase. However, we studied subjects around 63 years of age, the earlier study had subjects around 20 years of age.

A few notes should be made; the most important one being that almost all HRV is vagally mediated: both the fast beat-to-beat changes and the slower waves that may be riding on underlying blood pressure variations. As long as heart rate is in the vagal range, for humans below (120−0.6× age) (Karemaker et al., 1989), most variations in HR will, as first guess, mainly come from changes in vagal activity. That is not to say that the sympathetics play no role in HRV, their contribution can be found both in the underlying blood pressure variability and the longer-range variations in heart rate. These slower variations, to be observed over the course of minutes to hours, may influence both HR and BP at the same time, via central and peripheral mechanisms. In analysis techniques that aim at this “system complexity” it has become established that stable estimates may only be found when thousands of heart beats are incorporated, an order of magnitude requiring at least some 4 h observation. This is a requirement that is impractical for straightforward clinical monitoring. Although 4 h of data may become available in any patient on the monitor, a deterioration of condition should be signaled earlier than after 4 h.

In recent years the literature on HRV analysis methods has been reviewed for various areas of application. Rajendra Acharya et al. (2006) gave a more or less complete overview of methodologies that are applied, from time domain to frequency domain to non-linear analysis methods. Generally speaking, the main disadvantages of the latter are the large number of data required and the sensitivity to baseline shift and noise of some of the methods. In 2007 Maestri et al. (2007) reviewed the use of non-linear indices of HRV for CHF patients. Many were highly correlated to classical linear indices; only two (families of) analysis methods gave independent prognostic information, i.e., empirical mode decomposition and symbolic dynamics. We considered these not practical for our purpose. Again in 2009 Buccelletti et al. (2009) noted that techniques like power law (fractal) analysis or detrended fluctuation analysis were less practical in the prognosis of myocardial infarction patients than entropy directed methods in view of the number of required heart beats. In the present study we have, therefore, restricted our analysis to SampEn and MSE, being the most promising ones for our application. We looked at scale factors 6–10 for MSE, supported by a recent study by Ho et al. (2011) who had noticed that in CHF patients on β-blockade values of τ = 6 and up were insensitive to this therapy when used as predictors of mortality.

In short, the most reliable HRV parameters to early detect a patient's “slipping off” into a sympathetic state are those that indicate so-called vagal withdrawal, i.e., the disappearance of short-term variability and the loss of longer term “jumpiness” as shown by the decreased MSD_6−10_. These changes will occur even before heart rate goes up into a definite sympathetic region, where all vagal efferent traffic is silenced. The newer parameters like SampEn or MSE have no use here, at least not in the present group of patients who use β-blockers. A study by Batchinsky et al. (2009) has shown that SampEn can make a difference for triage in emergency care, even when only short recordings are available. Interestingly, the newly introduced parameter MSD_6−10_ seems to do a good job as well, detecting both the sympathetic shift and the difference between healthy controls and CHF-patients. This computes the “jumpiness” or rMSSD of coarse grained averages over 6–10 adjacent beats. This is not a non-linear parameter like MSE or SampEn, but one that is derived from the intermediate coarse grained series constructed for the computation of MSE. In that same vein we computed the variances of these series, which showed some promise in the controls-CHF comparison of Table 1, but failed to show a sympathetic shift (in Table 2).

**Figure 3:**
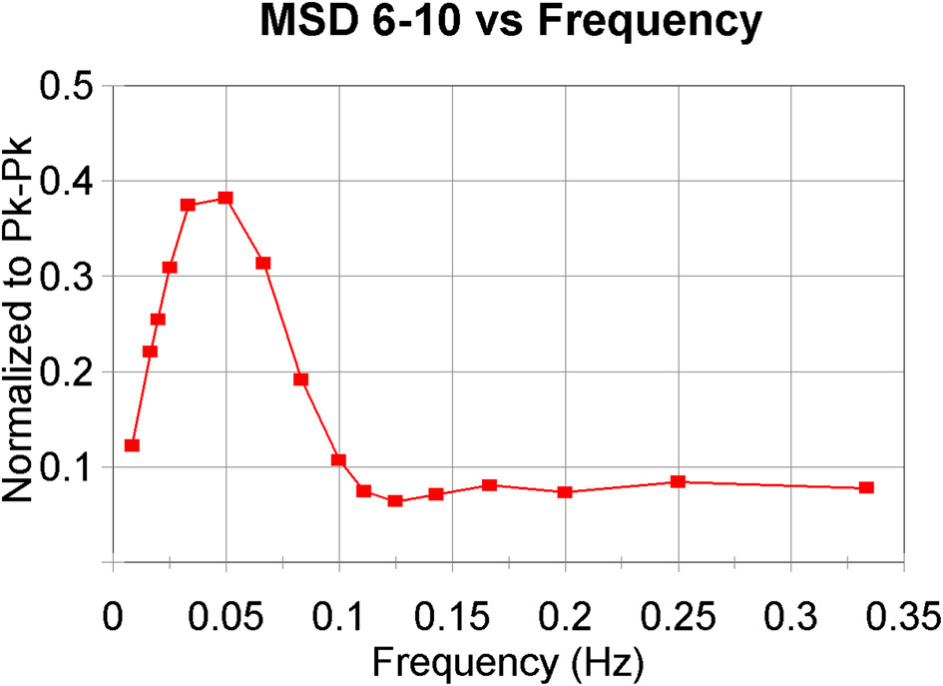
**Band-pass filter characteristics of MSD_6–10_**. The algorithm has been applied to model-generated beat-series with additional noise. A simplified model of baroreflex control has been used as in deBoer et al., 1987 to generate the intervals. Average heart period around 1000 ms. A modulating “respiratory” frequency was forced with periods from 3 to 120 s. The values for MDS_6–10_ have been normalized to the peak–peak amplitudes of the forced oscillations (Berntson et al., 2005).

rMSSD has peculiar properties, acting as a high-pass filter to the original heart rate signal. It has been proven (Berntson et al., 2005) that “classical” rMSSD captures the same frequency range as the HF band in frequency analysis does, roughly between 0.2 and 0.45 Hz. However, it is biased by the prevailing heart rate and is sensitive to lower frequencies as well. By extending the algorithm to progressively more coarse grained series of heart periods we have, with MSD_6−10_, constructed a combination of a low-pass filter (the coarse-graining process, cf. Figure 1) followed by a high-pass filter. Building on the earlier study into the rMSSD filter properties one may extrapolate that the number represented by MSD_6−10_ will favor frequencies between 0.02 and 0.07 Hz (i.e., 0.2/10 and 0.45/6 as 3dB points), thus mainly spanning the LF-band and slightly lower, as illustrated in Figure 3, the result of a simulation like in the Berntson study. It should be noted that these filter characteristics are dependent on the prevailing heart rate. In the present simulation, as in our study, we assumed an average heart rate of 60/min, 1 s intervals. This problem of scaling by heart rate is one that is omnipresent in MSE-studies, although very seldom mentioned.

### In conclusion

For patients on β-blockers only the gradual disappearance of short-term variability, as measured by traditional methods rMSSD and pIBI-50, proved a reliable indicator of a shift to a sympathetic state. The newly introduced MSD_6−10_—jumpiness in coarse grained beat series—shows some promise here as well. HRV analysis cannot work in clinical monitoring without taking HR-active medication into account; without β-blockade more parameters might be useful, notably SampEn and frequency analysis may carry useful information for the clinician. The best application for these measures is probably their use in intelligent monitoring, where the clinician is not bothered with the numbers and their intricacies, but just with the condition changes that are shown by analysis of HRV along with other vital parameters.

## Limitations

This study was conducted in a small number of relatively healthy CHF-patients: they had been stable on their medication before entering the study. The circumstances for patients admitted to the ICU for an acute cardiac condition may be quite different. Therefore, this study should be extended to real-life ICU-recordings and to larger groups of patients before definite conclusions about the usefulness of the present HRV-measures may be reached.

In a standard ICU-setting one would turn to the ECG-monitor for more accurate heart period detection than was possible here. The use of a 200 Hz sampled BP-recording limits the accuracy to ~5 ms, whereas normally at least 1 ms should be obtainable. Therefore, some measures that came out as rather insensitive now, like SampEn or MSE for low values of τ might perform better then. For “classical” MSE the present recordings were too short anyway, reason why we limited the τ (coarse graining parameter) to 10 rather than going to 20. At τ = 10 we have in a 10 min recording about 60 points for the coarse grained series. Since our τ-MSE curves reached stable levels for the data sets that we used, we considered this choice appropriate.

## Disclosures

None of the authors has any relevant disclosures to make. Ms. Zhang, PhD is the recipient of a post-doc training grant from the Department of Physiology.

### Conflict of interest statement

The authors declare that the research was conducted in the absence of any commercial or financial relationships that could be construed as a potential conflict of interest.

## Abbreviations

BMI: body mass index
CHF: chronic heart failure
HF: high frequency
HRV: heart rate variability
IBI: interbeat interval
ICU: Intensive Care Unit
LF: low frequency
MSD: multiscale root mean square of successive differences
MSE: Multi-Scale Entropy
MSV: multiscale variance
PVC: premature ventricular contraction
rMSSD: root mean square of successive differences of IBI's
pIBI-50: proportion of pairs of successive IBI's that differed by more than 50 ms
SampEn: sample entropy
SD-IBI: standard deviation of interbeat intervals
VLF: very low frequency.
